# Cadherin-17 as a target for the immunoPET of adenocarcinoma

**DOI:** 10.1007/s00259-024-06709-7

**Published:** 2024-04-16

**Authors:** Samantha Delaney, Outi Keinänen, Dennis Lam, Andrew L. Wolfe, Takao Hamakubo, Brian M. Zeglis

**Affiliations:** 1 Department of Chemistry, Hunter College of the City University of New York, 413 East 69th Street, New York, NY 10021 USA; 2 Ph.D. Program in Biochemistry, The Graduate Center of the City University of New York, New York, NY USA; 3 Department of Radiology, Memorial Sloan Kettering Cancer Center, New York, NY USA; 4 Department of Chemistry, University of Alabama at Birmingham, Birmingham, AL USA; 5 Department of Biological Sciences, Hunter College of the City University of New York, New York, NY USA; 6 Ph.D. Program in Biology (Molecular, Cellular, and Developmental Biology Sub-Program), The Graduate Center of the City University of New York, New York, NY USA; 7 Department of Pharmacology, Weill Cornell Medical College, New York, NY USA; 8 PhotoQ3 Inc., Tokyo, Japan; 9 Department of Radiology, Weill Cornell Medical College, New York, NY USA

**Keywords:** Adenocarcinoma, Pancreatic ductal adenocarcinoma, Cadherin-17, Zirconium-89, Positron emission tomography, Radioimmunoconjugate

## Abstract

**Purpose:**

Cadherin-17 (CDH17) is a calcium-dependent cell adhesion protein that is overexpressed in several adenocarcinomas, including gastric, colorectal, and pancreatic adenocarcinoma. High levels of CDH17 have been linked to metastatic disease and poor prognoses in patients with these malignancies, fueling interest in the protein as a target for diagnostics and therapeutics. Herein, we report the synthesis, *in vitro* validation, and *in vivo* evaluation of a CDH17-targeted ^89^Zr-labeled immunoPET probe.

**Methods:**

The CDH17-targeting mAb D2101 was modified with an isothiocyanate-bearing derivative of desferrioxamine (DFO) to produce a chelator-bearing immunoconjugate — DFO-D2101 — and flow cytometry and surface plasmon resonance (SPR) were used to interrogate its antigen-binding properties. The immunoconjugate was then radiolabeled with zirconium-89 (*t*_1/2_ ~ 3.3 days), and the serum stability and immunoreactive fraction of [^89^Zr]Zr-DFO-D2101 were determined. Finally, [^89^Zr]Zr-DFO-D2101’s performance was evaluated in a trio of murine models of pancreatic ductal adenocarcinoma (PDAC): subcutaneous, orthotopic, and patient-derived xenografts (PDX). PET images were acquired over the course of 5 days, and terminal biodistribution data were collected after the final imaging time point.

**Results:**

DFO-D2101 was produced with a degree of labeling of ~ 1.1 DFO/mAb. Flow cytometry with CDH17-expressing AsPC-1 cells demonstrated that the immunoconjugate binds to its target in a manner similar to its parent mAb, while SPR with recombinant CDH17 revealed that D2101 and DFO-D2101 exhibit nearly identical *K*_D_ values: 8.2 × 10^−9^ and 6.7 × 10^−9^ M, respectively. [^89^Zr]Zr-DFO-D2101 was produced with a specific activity of 185 MBq/mg (5.0 mCi/mg), remained >80% stable in human serum over the course of 5 days, and boasted an immunoreactive fraction of >0.85. In all three murine models of PDAC, the radioimmunoconjugate yielded high contrast images, with high activity concentrations in tumor tissue and low uptake in non-target organs. Tumoral activity concentrations reached as high as >60 %ID/g in two of the cohorts bearing PDXs.

**Conclusion:**

Taken together, these data underscore that [^89^Zr]Zr-DFO-D2101 is a highly promising probe for the non-invasive visualization of CDH17 expression in PDAC. We contend that this radioimmunoconjugate could have a significant impact on the clinical management of patients with both PDAC and gastrointestinal adenocarcinoma, most likely as a theranostic imaging tool in support of CDH17-targeted therapies.

**Supplementary Information:**

The online version contains supplementary material available at 10.1007/s00259-024-06709-7.

## Introduction

Cadherins are a superfamily of calcium-dependent proteins that mediate cell adhesion [1–3]. One member of this family — cadherin-17 (CDH17; liver intestine cadherin or LI-cadherin) — is typically expressed by intestinal epithelial cells [4, 5]. However, several types of adenocarcinoma cells overexpress CDH17, fueled by phosphorylation cascades within the ⍺_2_β_1_ integrin, Wnt/β-catenin, and Ras/Raf/MEK/ERK pathways [6, 7]. In recent years, CDH17 has attracted attention as a sensitive and specific biomarker for gastric, colorectal, and pancreatic cancers, outperforming other frequently used targets such as cytokeratin 20 (CK20) and the CDX2 gene [8]. High levels of CDH17 have also been linked to metastatic spread and poor prognoses in patients with several adenocarcinomas [9]. To wit, a clinicopathological study by Ge et al*.* found a strong correlation between the expression of CDH17 and the development of lymph node metastases in patients with gastric adenocarcinoma (*p* < 0.01, 166 patients: 40 at stage N0, 61 at stage N1, 37 at stage N2, and 28 at stage N3) [10]. Finally, the targeted inhibition of CDH17 has been shown to retard the growth of patient-derived pancreatic ductal adenocarcinoma (PDAC) xenografts in mice [11, 12]. Taken together, these data have spurred interest in CDH17 as a target for molecular therapies, including chimeric antigen receptor T-cells, antibody-drug conjugates, and agents for photodynamic therapy.

Monoclonal antibodies have long stood as promising platforms for the delivery of diagnostic and therapeutic radionuclides to tumor tissue [13, 14]. However, two phenomena have recently combined to generate a remarkable surge in the preclinical study and clinical deployment of radioimmunoconjugates. First, several radionuclides with favorable emission profiles and physical half-lives that align well with the pharmacokinetics of mAbs — most notably the positron-emitter zirconium-89 (^89^Zr, *t*_1/2_ ~ 3.3 days), the β-emitter lutetium-177 (^177^Lu, *t*_1/2_ ~ 6.6 days), and the α-emitter actinium-225 (^225^Ac, *t*_1/2_ ~ 9.9 days) — have become widely available to the nuclear medicine community [15]. Second, the desire for more precise and personalized therapies has driven the development of “theranostic” imaging probes that can help select patients likely to respond to targeted therapies as well as the creation of matched pairs of radiopharmaceuticals that can be used for both diagnosis and therapy [16, 17].

Herein, we report the synthesis, characterization, and *in vivo* validation of a ^89^Zr-immunoPET probe for the delineation of CDH17 expression in adenocarcinoma [18]. We have chosen to evaluate the performance of this imaging agent in murine models of PDAC for three reasons: (*i*) CDH17 is overexpressed in PDAC; (*ii*) other laboratories have developed and validated CDH17-targeted therapeutics for PDAC, suggesting that a CDH17-targeted immunoPET probe could be a valuable companion imaging agent; and (*iii*) there is an urgent need for novel theranostic tools for the clinical management of PDAC. Furthermore, while we appreciate the innovative nature of using genetically engineered immunoglobulins like minibodies as platforms for PET, the majority of extant CDH17-targeted therapeutics are based on intact IgGs. Therefore, we believe that a radioimmunoconjugate based on a full-length IgG would be best positioned to serve as a companion imaging agent in the clinic.

It is important to note that the immunoglobulin at the core of this investigation — D2101 — has already been leveraged to create radioimmunoconjugates, both an ^111^In-labeled mAb for immunoSPECT and a ^64^Cu-bearing minibody for PET [19, 20]. However, this work represents the first investigation in which the D2101 mAb has been paired with the radiometal ^89^Zr and, more importantly, the first time that a CDH17-targeted imaging agent has been evaluated in advanced murine models of PDAC (*i.e.*, orthotopic and patient-derived xenografts). In this study, [^89^Zr]Zr-DFO-D2101 exhibited excellent *in vivo* performance in mice bearing subcutaneous, orthotopic, and patient-derived xenografts of PDAC, consistently yielding images with substantial signal in the tumor and high tumor-to-healthy organ contrast. These encouraging data not only support the further preclinical exploration of the radioimmunoconjugate in other CDH17-expressing adenocarcinomas (*e.g.*, gastric adenocarcinoma) but also suggest that [^89^Zr]Zr-DFO-D2101 could have a role to play in the clinic as a companion theranostic imaging agent for CDH17-targeted therapies or (if labeled with a different radionuclide) a radioimmunotherapeutic.

## Materials and methods

### General

All reagents were purchased from Fisher Scientific (Waltham, MA, USA) unless otherwise noted. The CDH17-targeted antibody D2101 was produced by the Research Center of Advanced Science and Technology at The University of Tokyo according to published procedures [21]. *p*-SCN-Bn-DFO was purchased from Macrocyclics, Inc. (Plano, TX, USA). Protein measurements were determined with UV-Vis spectroscopy with a molar absorptivity of 2.1 × 10^5^ M^−1^ cm^−1^ and a molecular weight of 1.5 × 10^5^ Da. All water used was ultrapure (>18.2 MΩcm at 25 °C). MALDI-ToF mass spectrometry was performed by the Alberta Proteomics and Mass Spectrometry Facility (University of Alberta, Edmonton, AB, Canada). [^89^Zr]Zr^4+^ was provided by 3D Imaging (Little Rock, AR, USA).

### Flow cytometry

2.0 × 10^6^ CDH17-expressing AsPC-1 cells were aliquoted per sample and washed 3× with ice-cold PBS. Fifty microliters of either D2101 or DFO-D2101 (12 µg/mL) were added to the cells, incubated on ice for 30 min, and washed 3× with ice-cold PBS. Then, 50 µL of goat anti-human IgG Alexa-488 secondary antibody (12 µg/mL) were added to the cells, incubated on ice for 30 min in the dark, and washed 3× with ice-cold PBS. Following the washes, the cell pellets were resuspended in FACS buffer (PBS + 0.05% FCS + 2 mM EDTA). Samples were measured with a Becton-Dickinson Biosciences FACSCalibur Flow Cytometer, and the data was analyzed using FlowJo^TM^ Software. All suspensions were washed by centrifuging at 650 rcf for 2.5 min.

### Surface plasmon resonance

Per the manufacturer’s instructions, protein A was immobilized onto an activated carboxyl sensor using a Nicoya OpenSPR kit. D2101 and DFO-D2101 were diluted in running buffer (Cytiva HBS-P Buffer + PBS-BSA) and captured on the protein A. A multicycle kinetics experiment was performed by flowing solutions of 3.4, 11, 33, 100, and 300 nM recombinant CDH17 (in running buffer) over the sensor for 300 s with a Nicoya OpenSPR. Glycine-HCl (10 mM, pH 1.5) was used as a regeneration solution between each injection of antigen. Blank buffer runs were subtracted from the results and kinetics were determined using TraceDrawer.

### Radiolabeling and serum stability

DFO-D2101 (0.2 mg) was prepared in Chelex-treated PBS (0.5 mL, pH 7.4). [^89^Zr]Zr^4+^ supplied in oxalic acid was neutralized to pH 7.4 with 1 M Na_2_CO_3_, and the mAb was added to a volume of the pH-adjusted solution containing 37.0 MBq (1.00 mCi). The resulting solution was mixed thoroughly and allowed to react on an agitating ThermoMixer at 300 rpm for 30 min at 37 °C. The radiolabeling reaction was monitored via radio-instant thin-layer chromatography (iTLC) using glass-fiber silica-impregnated instant thin-layer chromatography paper (Pall Corp.; East Hills, NY, USA) and an eluent of 50 mM EDTA (pH 5.5). The iTLC plates were analyzed on an AR-2000 radio-iTLC plate reader with WinScan Software (Bioscan, Inc.; Washington, DC, USA). After the reaction, [^89^Zr]Zr-DFO-D2101 was purified via size-exclusion chromatography (PD-10 Column), and iTLC scans were performed again to verify the purity of the radioimmunoconjugate. To interrogate its stability, purified [^89^Zr]Zr-DFO-D2101 was placed in human serum, and quantitative iTLC measurements were acquired every 24 h for 5 days. All radio-iTLC measurements were performed in triplicate.

### Immunoreactivity measurements

The immunoreactive fraction of [^89^Zr]Zr-DFO-D2101 was determined via a bead-based assay performed as previously described [22]. In brief, 100 µL of HisPur^TM^ Ni-NTA magnetic beads were aliquoted per sample and placed on an Invitrogen^TM^ DynaMag^TM^-2 Magnetic Rack to isolate the beads from the storage buffer. The storage buffer was discarded, and the beads were washed 2× with PBS supplemented with 0.05% Tween 20 and 50 mM imidazole (PBS-T). Five micrograms of recombinant CDH17 antigen were added to the beads, and the resulting mixture was agitated and allowed to react on a ThermoFisher Scientific Tube Revolver Rotator at room temperature for 30 min. Following this incubation, the beads were washed 2× more with PBS-T, and 1 ng of [^89^Zr]Zr-DFO-D2101 was added to each sample. The tubes were then placed back on the rotator and allowed to incubate. After 30 min, the tubes were placed on the magnetic rack, and the supernatants were collected in separate tubes. Subsequently, two final washes of the beads with PBS-T were performed and collected in separate tubes. Two control cohorts — a blocking group that received 5 µg of cold DFO-D2101 and a control group that used no CDH17 — were used alongside the experimental cohort. The amount of radioactivity in each sample was analyzed on a ^89^Zr-calibrated gamma counter, and the activities (counts/minute) were background- and decay-corrected to the start of the run. The immunoreactive fraction was determined by dividing the amount of radioactivity associated with the beads by the total radioactivity in the beads, supernatant, and wash samples.

### Subcutaneous xenografts

Five-to-seven-week-old athymic nude mice were obtained from The Jackson Laboratory (Bar Harbor, ME, USA) and allowed to acclimatize for 1 week before inoculation. The animals were housed in ventilated cages and given food and water *ad libitum*. Prior to injection, the mice were anesthetized by inhalation of 2% isoflurane/oxygen gas mixture (Baxter Healthcare; Deerfield, IL, USA), and the injection site was sanitized with ethanol. Tumors were induced in the right shoulder via the subcutaneous injection of 5 × 10^6^ AsPC-1 cells in a 1:1 mixture of media:MatriGel (Corning Life Sciences; Corning, NY, USA). To ensure homogenous tumors, the implantation suspension was thoroughly mixed prior to each inoculation. Tumors reached ideal size for PET imaging and biodistribution studies after 3 weeks.

### Orthotopic models and bioluminescence imaging

Five-to-seven-week-old athymic nude mice were obtained from The Jackson Laboratory (Bar Harbor, ME, USA) and allowed to acclimatize for 1 week before inoculation. The animals were housed in ventilated cages and given food and water *ad libitum*. An incision was made in the left flank, and 5 × 10^6^ AsPC-1_*luc*_ cells were implanted into the pancreas of each animal. All animals were monitored during the post-operation window, and the sutures were removed 2 weeks after surgery. Bioluminescent imaging was used to monitor tumor growth following implantation via the intraperitoneal injection of 100 µL of 30 mg/mL firefly D-luciferin in PBS. The mice were then anesthetized via inhalation of 2% isoflurane/oxygen gas mixture and, at 15 min post-injection, were imaged on an IVIS^®^ Spectrum-CT instrument in the prone position. All bioluminescent images were analyzed with Living Image^®^. Tumors reached ideal size for PET imaging and biodistribution studies after 3 weeks.

### Immunohistochemistry and autoradiography of orthotopic xenografts

Tumor tissue embedded in the pancreas was harvested and flash-frozen in a mold with optimal cutting temperature (OCT) compound. The mold was cut on an Avantik Cryostat-Microtome at −18 °C into 10-µm slices and fixed onto microscope slides with acetone.

Histology was performed on the tissues with an Abcam Hematoxylin and Eosin Staining Kit. The slides were first rinsed with PBS 3× to remove excess OCT and then dipped in the hematoxylin stain for 2 min. After 3 washes with deionized H_2_O, the slides were dipped in bluing reagent for 15 s and washed 2× more with deionized H_2_O. Following the washes, the slides were quickly dipped in 100% ethanol, air dried, and stained with eosin for 2 min. Then, after 3 washes with 100% ethanol, DPX Mounting Media was added to slides, and the slides were covered with glass cover slips and sealed. The slides were imaged by the Memorial Sloan Kettering Cancer Center Imaging and Image Analysis Core.

Slides for autoradiography were wrapped in cellophane and placed in a FisherBioTech Autoradiography Cassette with a phosphor imaging plate atop the slides. The cassette was incubated in the dark for 48 h, after which the plate was imaged with a GE Typhoon FLA 7000 instrument and analyzed with Typhoon FLA 7000 Control Software.

### Immunofluorescence and confocal imaging of patient-derived tissue

Ten tissue samples from PDAC patients were obtained from the Memorial Sloan Kettering Cancer Center Anti-Tumor Assessment Core and stained with a recombinant CDH17-binding mAb (Abcam; ab109190). The paraffin-fixed formalin-embedded slides were deparaffinized with a mixture of xylenes and ethanol, and trypsin buffer was used as an antigen-retrieval solution. The slides were then placed in a Coplin jar with 5% donkey serum to block any non-specific binding sites and washed 3× in Coplin jars filled with PBS. A 5 µg/mL solution of the CDH17-binding mAb was prepared and pipetted directly onto the tissues, and the slides were allowed to incubate in a humidity chamber for 1 h. The slides were washed 3× in Coplin jars with fresh PBS, and a secondary antibody solution (goat anti-human IgG Alexa-488 + DAPI) was pipetted onto the tissues and allowed to incubate in a humidity chamber for 1 h. After 3 more washes with PBS, Fluoroshield^TM^ was added to the slides, and the slides were covered with glass cover slips and sealed. The slides were imaged on a Nikon Eclipse Ti Confocal Microscope. Of the ten samples imaged, three — 38a (stage IIB), 46a (stage IB), and 56b (stage IV) — were chosen to be grown in mice as PDXs. For comparison, an AsPC-1 tumor was also grown, cryogenically frozen, sliced on a microtome, and stained with the CDH17-binding mAb.

38a, 46a, and 56b tissue were each subcutaneously implanted in NSG mice obtained from The Jackson Laboratory. Once the tumors had reached a size ideal for passaging, each tumor type was implanted in 4–5 additional mice. In these mice, the PDX tumors reached ideal sizes for imaging in ~ 2 weeks.

### PET imaging

PET images were obtained using either a microPET Focus 120 small animal scanner or an Inveon PET-CT small animal scanner (Siemens Medical Solutions; Malvern, PA, USA). Once the AsPC-1, AsPC-1_*luc*_, or PDX tumors reached an appropriate size for experiments, the animals’ veins were dilated via heating, the skin was sterilized with ethanol, and [^89^Zr]Zr-DFO-D2101 was administered via intravenous tail vein injection. After injection, all mice (*n* = 4 per cohort) underwent static scans every 24 h for 6 days, and at least 4 × 10^7^ counts were obtained from each scan. The counting rates in the reconstructed images were converted to activity concentrations (percent injected dose per gram of tissue (%ID/g)) using a system calibration factor derived from the imaging of a mouse-sized water-equivalent phantom containing ^89^Zr. Maximum intensity projection (MIP) images were generated from 3-dimensional ordered subset expectation maximum reconstruction (3D-OSEM). The images were analyzed with ASIPro VM^TM^ (Concorde Microsystems; Knoxville, TN, USA) or Inveon Research Workplace Software.

### Biodistribution experiments

After the final time point of each imaging study, the animals were euthanized by CO_2(g)_ asphyxiation followed by cervical dislocation. Selected organs were harvested, rinsed in water, dried, weighed, and quantified on a ^89^Zr-calibrated PerkinElmer Wizard^2^ γ-counter. The counts/minute value for each tissue was background- and decay-corrected to the start of the activity measurement. The %ID/g value for each sample was then calculated by normalization to the total injected activity.

## Results

### Bioconjugation and structural characterization of DFO-D2101

The acyclic linear siderophore desferrioxamine (DFO) is currently the “gold-standard” chelator for [^89^Zr]Zr^4+^ because its sextet of oxygen donors provides the oxophilic radiometal with a thermodynamically and kinetically stable coordination environment [23]. In this case, DFO-D2101 was prepared according to published protocols via the stochastic attachment of *p*-SCN-Bn-DFO to lysines on the surface of the immunoglobulin [24]. Matrix-assisted laser desorption/ionization time-of-flight mass spectrometry (MALDI-ToF) revealed a degree-of-labeling (DOL) of ~ 1.1 DFO/mAb, and size exclusion chromatography showed that no aggregates had been formed due to the conjugation of the chelator (Figures S1-S2).

**Fig. 1:**
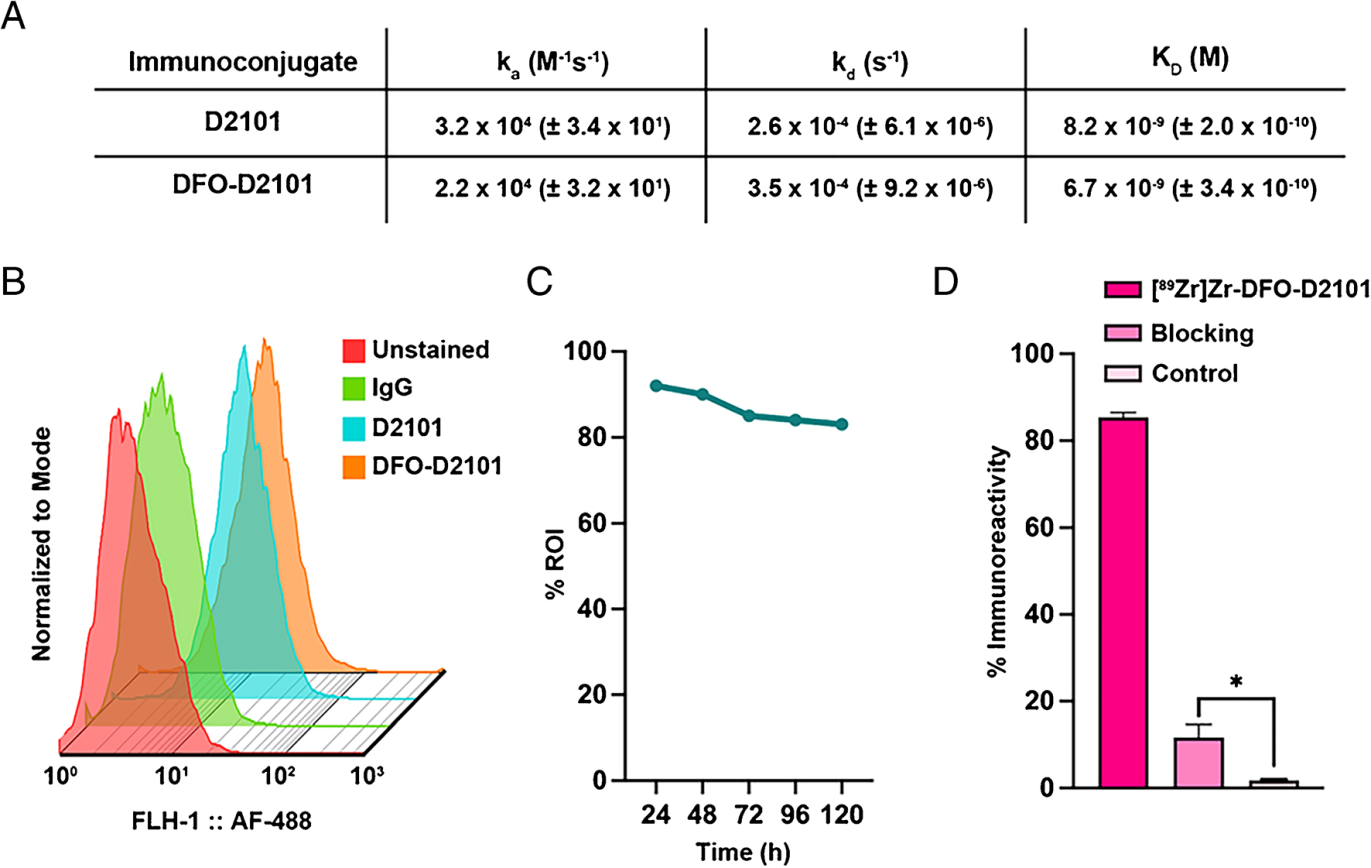
**A** Binding parameters of D2101 and DFO-D2101 for CDH17 as determined via surface plasmon resonance. **B** Flow cytometry data for D2101 and DFO-D2101 with CDH17-expressing AsPC-1 cells. **C** Longitudinal stability study of [^89^Zr]Zr-DFO-D2101 in human serum. **D** Immunoreactivity assay for [^89^Zr]Zr-DFO-D2101 performed using CDH17-coated magnetic beads

A pair of *in vitro* assays were employed to verify that the attachment of DFO to D2101 did not adversely affect the antibody’s ability to bind CDH17. First, flow cytometry experiments were performed with CDH17-expressing AsPC-1 cells, and the near-identical shift patterns suggested that D2101 and DFO-D2101 bind CDH17 in a similar manner. Subsequently, surface plasmon resonance was employed to determine the quantitative binding parameters of each construct for CDH17. D2101 and DFO-D2101 displayed nearly identical *K*_D_ values — 8.2 × 10^−9^ and 6.7 × 10^−9^ M, respectively — as well as strikingly similar *k*_a_ and *k*_d_ rates (Figure 1A–B, Figure S3).

### Synthesis and evaluation of [^89^Zr]Zr-DFO-D2101

DFO-D2101 was radiolabeled with [^89^Zr]Zr^4+^ according to published protocols to produce [^89^Zr]Zr-DFO-D2101 [25]. Radio-instant thin-layer chromatography (radio-iTLC) and radio-size exclusion chromatography HPLC (radio-SEC) were used to monitor the reaction and revealed that the radioimmunoconjugate was synthesized in >97% radiochemical yield, >99% radiochemical purity, and a specific activity of ~185 MBq/mg (~ 5.0 mCi/mg) (Figure S4). To interrogate the stability of the radioimmunoconjugate, [^89^Zr]Zr-DFO-D2101 was incubated in human serum at 37 °C, and radio-iTLC measurements taken every 24 h revealed that the radioimmunoconjugate remained >80% and >71% stable in human and mouse serum, respectively, over the course of 5 days (Figure 1C, Figure S5). Subsequently, an immunoreactivity assay with CDH17-coated magnetic beads revealed that [^89^Zr]Zr-DFO-D2101 boasted an immunoreactive fraction of >0.85 (Figure 1D). Taken together, these data indicated that we had produced a robust and stable radioimmunoconjugate with unperturbed antigen-binding properties compared to its parent mAb.

### In vivo evaluation: subcutaneous, orthotopic, and patient-derived xenografts

In order to develop a comprehensive view of [^89^Zr]Zr-DFO-D2101’s *in vivo* performance, the radioimmunoconjugate was evaluated in a trio of murine models of PDAC: subcutaneous, orthotopic, and patient-derived xenografts. For the subcutaneous xenografts, we employed CDH17-expressing AsPC-1 human PDAC cells acquired from ATCC. For the orthotopic xenografts, we developed a luciferase-expressing variant of the AsPC-1 cell line: AsPC-1_*luc*_. And for the PDXs, patient-derived PDAC tissue was provided by the Anti-Tumor Assessment Core of Memorial Sloan Kettering Cancer Center.

**Fig. 2:**
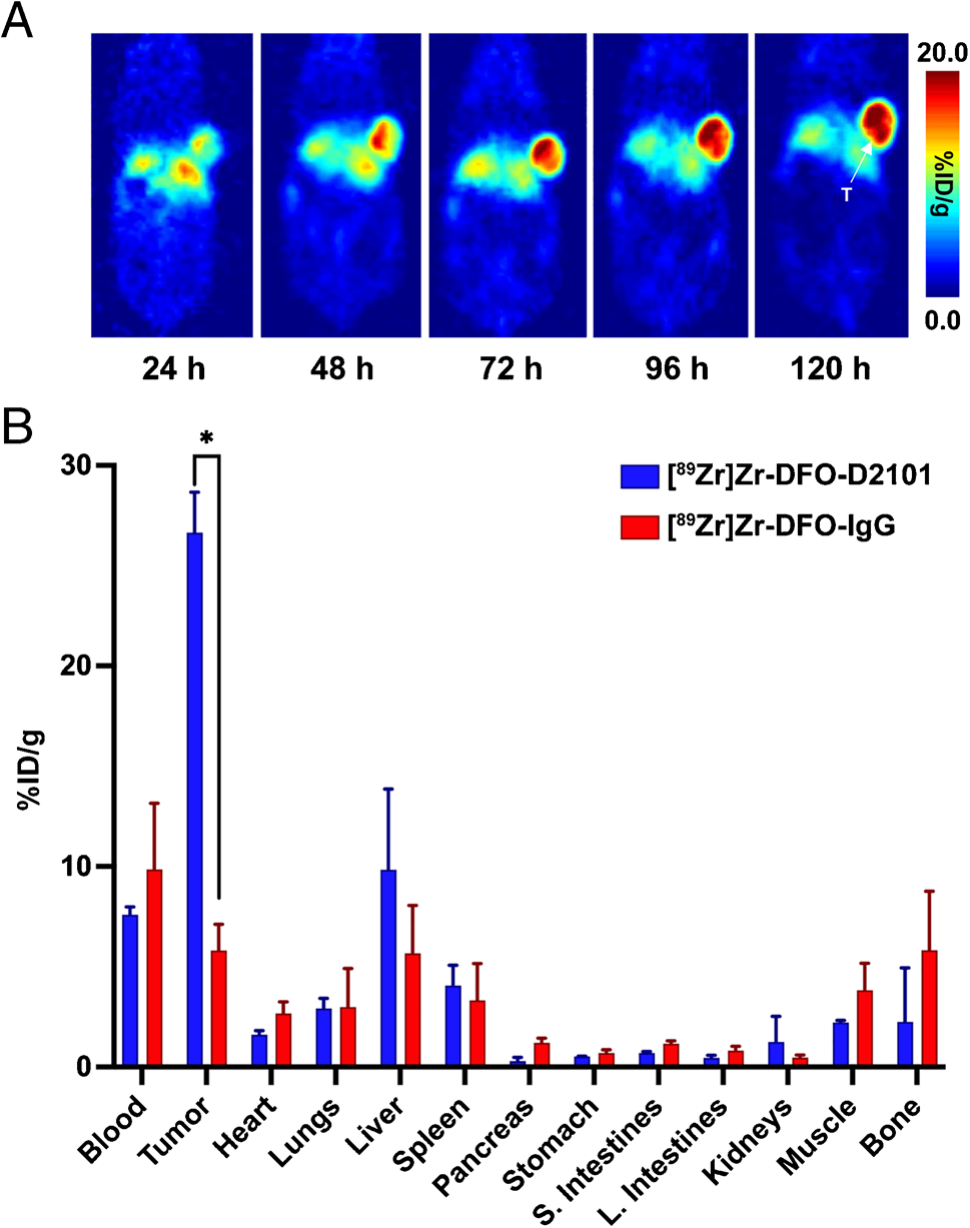
**A** Representative coronal PET images obtained following the administration of [^89^Zr]Zr-DFO-D2101 to athymic nude mice bearing subcutaneous AsPC-1 PDAC xenografts (T, tumor). **B** *Ex vivo* biodistribution data collected 120 h after the administration of [^89^Zr]Zr-DFO-D2101. [^89^Zr]Zr-DFO-IgG was used as a control. Statistical significance was determined via a two-tailed *t* test with a Welch’s correction using GraphPad Prism: *p < 0.05

In our pilot study, athymic nude mice (*n* = 5) were inoculated with AsPC-1 cells in the right shoulder, and tumors reached ~ 100 mm^3^ in ~ 3 weeks. 3.7 MBq (100 µCi) of [^89^Zr]Zr-DFO-D2101 was administered in 100 µL PBS via intravenous tail injection, and static PET scans were acquired every 24 h for 5 days. The PET images clearly delineated tumor tissue as early as 24 h post-injection, and the tumor-to-background image contrast improved throughout the course of the study (Figure 2A, Figure S6). Amongst healthy tissues, the liver exhibited the highest uptake, though it remained far below that of the tumor. After the final imaging time point, the animals were euthanized, and quantitative biodistribution data were collected. As expected, the tumor boasted the highest activity concentration (26.6 ± 2.0 %ID/g), the liver was the healthy tissue with the highest degree of uptake (9.8 ± 4.0 %ID/g), and all other healthy tissues exhibited accretion levels <5.0 %ID/g (Figure 2B). The activity concentration in the blood decreased gradually over the course of the experiment to 7.6 ± 0.4 %ID/g at 120 h p.i. This behavior is typical of full-length radioimmunoconjugates, and while it can be ameliorated somewhat by using smaller format antibody fragments as scaffolds, such a change often comes at the cost of reduced accretion in the target tissue. Importantly, the specificity of the probe was underscored by the reduced tumoral activity concentrations observed in a pair of control cohorts: mice injected with a non-specific, isotype-control radioimmunoconjugate ([^89^Zr]Zr-DFO-IgG; Figure S7, Table S1) and mice administered [^89^Zr]Zr-DFO-D2101 admixed with an excess of unmodified D2101 (~ 400 µg/mouse) (Figure S8, Table S1).

**Fig. 3:**
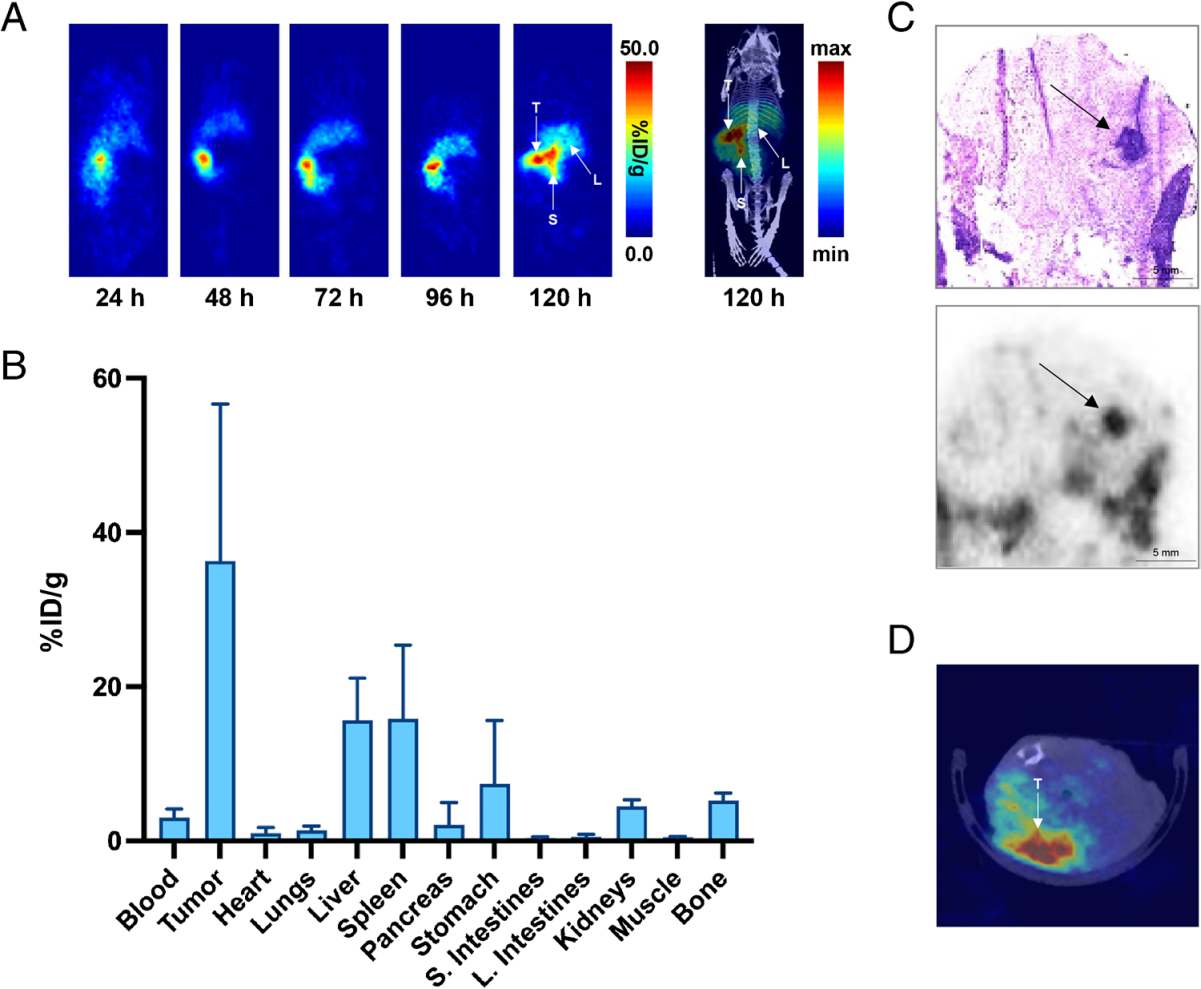
**A** Representative coronal PET images and a PET-CT maximum intensity projection obtained following the administration of [^89^Zr]Zr-DFO-D2101 to athymic nude mice bearing orthotopic AsPC-1_*luc*_ PDAC xenografts (T, tumor; S, spleen; L, liver). **B** *Ex vivo* biodistribution data collected 120 h after the administration of [^89^Zr]Zr-DFO-D2101. **C** CDH17 immunohistochemical staining (top) and autoradiographic (bottom) images of pancreas tissue excised 120 h after the administration of [^89^Zr]Zr-DFO-D2101 (arrows denote tumor lesions). **D** Transverse PET-CT image acquired 120 h after the administration of [^89^Zr]Zr-DFO-D2101 (T, tumor; same mouse as in panel **A**)

Encouraged by these results, we next turned to more clinically relevant animal models. We first interrogated the performance of [^89^Zr]Zr-DFO-D2101 in mice bearing orthotopic PDAC xenografts. To this end, a luciferase-expressing variant of AsPC-1 cells — AsPC-1_*luc*_ — was generated via transduction and then surgically implanted into the parenchyma of the pancreas of nude mice (*n* = 4) (Figures S9-S10). The growth of the tumors was monitored via bioluminescence imaging, and the xenografts reached a suitable size for imaging after ~ 3 weeks (Figure S11). At this point, 2.96 MBq (80 µCi) of [^89^Zr]Zr-DFO-D2101 was administered in 100 µL PBS via the lateral tail vein, and static PET scans were acquired every 24 h for 5 days. In the resulting images, the tumor tissue was clearly visible as early as 24 h post-injection, and both the tumoral activity concentration and the tumor-to-background contrast increased over the course of the experiment (Figure 3A, Figure S12). Biodistribution data collected after the final imaging time point confirmed the PET data. At 120 h post-injection, the tumor easily boasted the highest activity concentration: 36.3 ± 20.3 %ID/g. Healthy tissues generally displayed uptake levels <10 %ID/g, with the liver (15.6 ± 5.5 %ID/g) and spleen (15.9 ± 9.5 %ID/g) the only exceptions (Figure 3B, Table S2). The *ex vivo* analysis of excised pancreatic tissue via hematoxylin and eosin staining and autoradiography confirmed the selective uptake [^89^Zr]Zr-DFO-D2101’s in tumor cells embedded within the organ (Figure 3C).

**Fig. 4:**
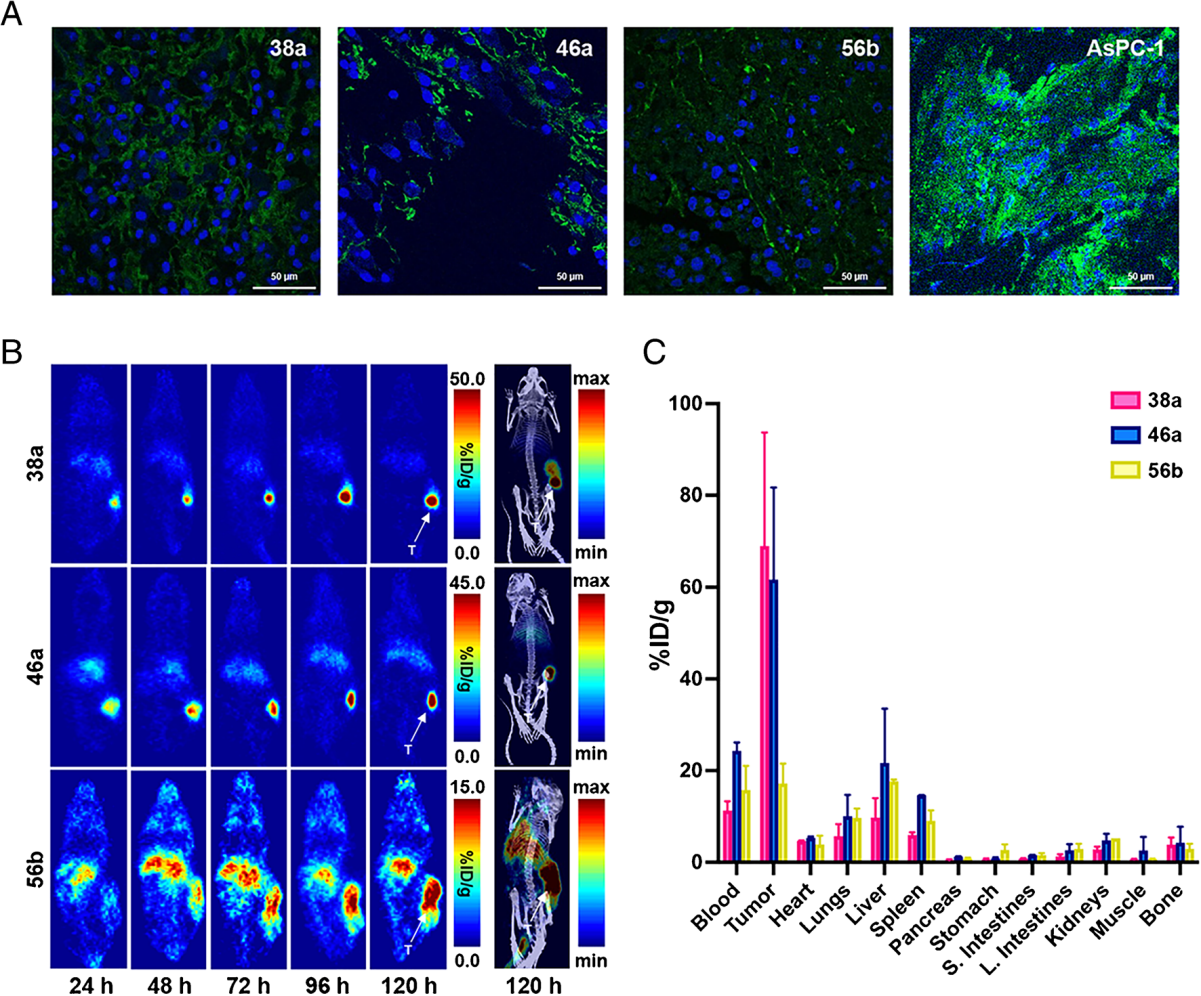
**A** Confocal microscopy images of a trio of patient-derived PDAC tissues — 38a, 46a, and 56b — following immunofluorescence staining for CDH17. Blue = DAPI; Green = staining for CDH17 visualized via goat anti-human IgG Alexa-488. A CDH17-expressing AsPC-1 tumor was also frozen, cut, stained for CDH17, and imaged. **B** Representative coronal PET images and a PET-CT maximum intensity projection obtained following the administration of [^89^Zr]Zr-DFO-D2101 to NSG mice bearing patient-derived PDAC tumors (T, tumor)*.* **C** *Ex vivo* biodistribution data collected 120 h after the administration of [^89^Zr]Zr-DFO-D2101

Finally, [^89^Zr]Zr-DFO-D2101 was evaluated in mice bearing patient-derived PDAC xenografts. Immunofluorescence staining of ten PDAC tissue samples obtained from the Memorial Sloan Kettering Cancer Center Anti-Tumor Assessment Core produced broadly similar results. Three models — 38a (stage IIB), 46a (stage IB), and 56b (stage IV) — were selected to be grown in NSG mice (*n* = 4 for each model) (Figure 4A, Figure S13, Table S3). The subcutaneous PDXs reached appropriate sizes for imaging in ~ 2 weeks, at which point 2.96 MBq (80 µCi) of [^89^Zr]Zr-DFO-D2101 was administered in 100 µL PBS via intravenous tail vein injection. Subsequently, PET scans were acquired every 24 h for 5 days, and on the last day, the animals were euthanized to facilitate the collection of biodistribution data. The mice bearing the 38a and 46a xenografts produced the PET images with the highest tumor-to-background contrast and exhibited the highest tumoral activity concentrations — 69.1 ± 24.7 and 61.7 ± 20.0 %ID/g, respectively — of any of the *in vivo* experiments performed in this investigation (Figure 4B–C, Figure S14). Somewhat surprisingly, these two models also yielded strikingly low off-target uptake values, below even those of the mice bearing subcutaneous AsPC-1 xenografts. Interestingly, while the 56b tissue produced very similar *in vitro* staining data to that of the other two PDX samples, the mice bearing the 56b PDXs displayed significantly reduced accretion of [^89^Zr]Zr-DFO-D2101 in the tumor (*i.e.*, 17.2 ± 4.3 %ID/g) as well as higher uptake in several healthy tissues (*e.g.*, 17.7 ± 0.4 and 15.8 ± 5.3 %ID/g in the liver and blood, respectively) (Table S4-S5). That said, the tumor tissue is still clearly visualized in these PET images, and the activity concertation in the tumor far exceeds that which can be attributed to the enhanced permeability and retention (EPR) effect alone.

## Discussion

The clinical promise of any radiotheranostic ultimately boils down to two issues: (1) the *in vivo* performance of the probe and (2) the diagnostic or therapeutic value of the target. With respect to the former, these data clearly illustrate that [^89^Zr]Zr-DFO-D2101 is a robust and effective probe for the visualization of CDH17-expressing PDAC tissue. While the initial experiments in mice bearing subcutaneous xenografts were promising, we understand that subcutaneous xenografts have significant drawbacks as tumor models. As a result, we next evaluated the radioimmunoconjugate in mice bearing two models of PDAC — orthotopic AsPC-1_*luc*_ xenografts and subcutaneous patient-derived xenografts — that are better recapitulations of human disease. The experiments in these mice reinforced the promise of [^89^Zr]Zr-DFO-D2101, as the probe consistently produced images with high tumoral uptake and excellent tumor-to-healthy organ contrast. Somewhat surprisingly, but nonetheless exciting, the “best” images were obtained in mice bearing a pair of PDAC PDXs (38a and 46a) — hopefully a harbinger of the clinical promise of the agent. At present, it is unclear why the uptake in the 56b PDX was lower than that in the other two xenografts, especially since the expression of CDH17 appears similar in the immunofluorescence images. Differences in perfusion and density are most likely responsible, though the fact that the parent tumor of 56b had been treated while the parent tumors of the other two PDXs were treatment-naïve may also be relevant.

With respect to the second aforementioned criterion, we contend that CDH17’s promise as an oncologic target is clear: it is overexpressed by a variety of adenocarcinomas, is associated with metastatic spread and poor prognosis, and has outperformed several other biomarkers as a diagnostic tool. The most exciting clinical application of [^89^Zr]Zr-DFO-D2101 may be as a companion theranostic imaging agent for CDH17-targeted therapies, as the non-invasive nature of immunoPET offers distinct advantages over extant methods (*i.e.*, biopsy) for the annotation of CDH17 expression by tumor tissue. CDH17 has already proven to be a viable target for therapy. In 2020, for example, Lum et al*.* combined the CDH17-targeting mAb ARB102 with the near-infrared fluorophore IR700 to create a photodynamic therapy agent that inhibited the growth of AsPC-1 PDAC xenografts in mice [11]. More recently, Ma et al*.* appended the toxin PE38 to a CDH17-targeting V_HH_ to create a nanobody-drug conjugate that inhibited the growth of PDAC PDXs *in vivo* [12]. These data suggest that CDH17 may have promise as a target for radiopharmaceutical therapy as well. Finally — yet perhaps most excitingly — a collaborative effort between the University of Pennsylvania and Chimeric Therapeutics recently yielded an approach to CAR T-cell therapy predicated on targeting CDH17. In preclinical models, this strategy successfully inhibited the growth of several different neuroendocrine and gastrointestinal solid tumors. The FDA has recently approved an investigational new drug application for a phase 1/2 trial of this technology in patients with CDH17-expressing malignancies (NCT06055439) [26].

PDAC — while an excellent context for the preclinical evaluation of [^89^Zr]Zr-DFO-D2101 — may not even be the indication in which the radioimmunoconjugate could be most valuable. Indeed, even the most cursory perusal of the literature reveals that several adenocarcinomas abundantly express CDH17. Going forward, gastric and colorectal adenocarcinomas may be the indications in which [^89^Zr]Zr-DFO-D2101 will have the most utility, as patients with each have been shown to express CDH17, and both cancers are plagued by a lack of reliable radiotheranostics [27].

Finally, it is critical to address one unavoidable limitation of the study: D2101 does not bind murine CDH17, and thus, the PET imaging studies described were obtained in an environment in which the binding of the radioimmunoconjugate to antigen expressed by healthy tissues cannot interfere with its accretion in the tumor. Importantly, we do not believe that the endogenous expression of CDH17 will be an impediment to the use of [^89^Zr]Zr-DFO-D2101 in patients with PDAC, as healthy pancreas expresses much lower levels of the antigen [28]. Furthermore, the lack of CDH17 expression by healthy stomach tissue similarly suggests that the probe could be valuable in patients with stomach and gastric cancer, but higher levels of expression in the colon may portend challenges in the context of colorectal adenocarcinoma [29, 30]. As we move forward with this work, we are pursuing the creation of a genetically engineered mouse model that expresses human CDH17 and thus will provide a more accurate backdrop for *in vivo* imaging and therapy experiments.

## Conclusions

In this investigation, we have clearly demonstrated the efficacy of [^89^Zr]Zr-DFO-D2101 as a tool for the non-invasive delineation of CDH17-expressing PDAC in subcutaneous, orthotopic, and patient-derived xenograft models of the disease. We believe that these data suggest that [^89^Zr]Zr-DFO-D2101 could be a valuable agent for the clinical immunoPET of CDH17-positive PDAC and other cancers that express the antigen. We are currently working to explore the performance of [^89^Zr]Zr-DFO-D2101 in murine models of stomach and gastric adenocarcinoma, to interrogate the potential of CDH17 as a target for radioimmunotherapy, to create smaller format CDH17-targeted probes that offer more rapid pharmacokinetic profiles, and to develop an improved mouse model for the study of CDH17-targeted probes.

### Supplementary Information

Below is the link to the electronic supplementary material.

Supplementary file1 (PDF 7804 KB)

## Data Availability

The data are available upon request.
