# Supplementary material for: Cadherin-17 as a target for the immunoPET of adenocarcinoma
Source: Eur J Nucl Med Mol Imaging. 2024 Apr 16;51(9):2547–57. doi: 10.1007/s00259-024-06709-7 (PMC11223962; doi:10.1007/s00259-024-06709-7)
Supplement: Supplementary file 1 — Supplementary file1 (PDF 7804 KB) [file 259_2024_6709_MOESM1_ESM.pdf]

Supplemental Information

Cadherin-17 as a Target for the ImmunoPET of Adenocarcinoma

Samantha Delaney[1,2,3], Outi Keinänen[4], Dennis Lam[5], Andrew L. Wolfe[2,5,6,7], Takao Hamakubo[8], Brian M. Zeglis[1,2,3,9]

[1]Department of Chemistry, Hunter College of the City University of New York, New York, NY, USA

[2]Ph.D. Program in Biochemistry, The Graduate Center of the City University of New York, New York, NY, USA

[3]Department of Radiology, Memorial Sloan Kettering Cancer Center, New York, NY, USA

[4]Department of Chemistry, University of Alabama at Birmingham, Birmingham, AL, USA

[5]Department of Biological Sciences, Hunter College of the City University of New York, New York, NY, USA

[6]Ph.D. Program in Biology (Molecular, Cellular, and Developmental Biology Sub-Program), The Graduate Center of the City University of New York, New York, NY USA

[7]Department of Pharmacology, Weill Cornell Medical College, New York, NY, USA

[8]PhotoQ3 Inc., Tokyo, Japan

[9]Department of Radiology, Weill Cornell Medical College, New York, NY, USA

Corresponding Author: Brian M. Zeglis; 413 East 69th Street, New York, NY, 10021; Phone: 212-896-0433; E-mail: bz102@hunter.cuny.edu

Keywords: Adenocarcinoma, pancreatic ductal adenocarcinoma, cadherin-17, zirconium-89, positron emission tomography, radioimmunoconjugate

SUPPORTING METHODS

Synthesis of DFO-D2101

D2101 was prepared in filtered Chelex-treated PBS (pH 7.4) and diluted to a final concentration of 0.5-1.0 mg/mL. Small aliquots of 0.1 M Na_2CO_3 were used to increase the pH of the mAb solution to 8.8-9.0, and *p*-SCN-Bn-DFO in DMSO (20 equiv., 12.5 mg/mL) was slowly added to the solution and thoroughly mixed. The reaction was incubated for 1 h on an agitating ThermoMixer at 37 ˚C. Following the reaction, the mAb was purified with size-exclusion chromatography (PD-10 Column; GE Healthcare; Chicago, IL, USA) and then concentrated with a 2 mL Amicon Ultra centrifugal filter with a 50 kDa molecular weight cut-off (MilliporeSigma).

Cell Culture

All cell lines used were obtained from American Type Culture Collection (Manassas, VA, USA) and grown under sterile conditions. The human pancreatic ductal adenocarcinoma cell line AsPC-1 was maintained in Roswell Park Memorial Institute media supplemented with 10% heat-inactivated fetal calf serum, 10 mM HEPES with 100 units/mL penicillin and 100 units/mL streptomycin, 1 mM sodium pyruvate, 2 mM L-glutamine, 1.5 g/L sodium bicarbonate, and 4.5 g/L-glucose. Human Embryo Kidney-293T cells were maintained in Dulbecco's Modified Eagle's media supplemented with 10% heat-inactivated fetal calf serum, 1 mM sodium pyruvate, 2 mM L-glutamine, 100 units/mL penicillin, and 100 units/mL streptomycin. All media were purchased from the Media Preparation Core at Memorial Sloan Kettering Cancer Center.

Generation of AsPC-1$_{luc}$ Cells

Luciferase-expressing AsPC-1 cells were generated via a plasmid transfection. To begin, the pHIV-iRFP720-EsA-Luc plasmid was obtained from AddGene in a bacterial stab, streaked onto a prepared Luria Broth agar plate supplemented with ampicillin, and allowed to incubate overnight at 37 ˚C. The viral packaging (pMD2.G) and envelope (psPAX2) plasmids were prepared in DH5α competent bacterial cells. 1 ng/µL of each plasmid was added to separate aliquots of the cells and incubated on ice for 15 min. The cells were then heat shocked on a ThermoMixer set to 42 ˚C for exactly 45 s. After, 1 mL of Luria Broth was added to the cells, and the tubes were incubated for 1 h at 225 rpm. Following the incubation, the cells were centrifuged at 3,000 rpm for 5 min,

resuspended in 100 µL Luria Broth, streaked onto Luria Broth agar plates supplemented with ampicillin, and allowed to incubate overnight at 37 ˚C.

One of the resulting colonies from each of the plasmids' plates were picked with a sterile pipet tip, placed in Luria Broth supplemented with ampicillin in 5 mL cell culture tubes, and allowed to incubate for 1 h at 37˚C and 225 rpm. Subsequently, the solutions were transferred to 125 mL Erlenmeyer flasks and were left for overnight incubation under the same conditions. The solutions were then centrifuged, the pellets were resuspended, and the DNA was isolated from the bacterial cells using a QIAGEN Plasmid *Plus* Midi Sample kit. All DNA concentrations were determined using a NanoDrop™ One^{C} UV-Vis spectrophotometer.

Lentivirus generation and transfection began by preparing the DNA solutions with Opti-MEM™ and a Lipofectamine 3000 Transfection Reagent kit. 6 µg of the transfer luciferase plasmid, 4.5 µg of the envelope plasmid, and 1.5 µg of the packaging plasmid were used to create DNA-lipid complexes, which were then pipetted to human embryonic kidney 293T cells in culture. Media changes of the 293T cells were performed at 6, 24, and 48 h after the introduction of the DNA-lipid complexes. At 72 h, an EVOS® FL Auto Imaging System was used to confirm the successful transfection of the iRFP-containing luciferase plasmid to the 293T cells (*Figure S1*). The media of the 293T cells was then harvested, filtered at 45 µm (Millex® PVDF Syringe Filter, Cat. No. SLHVR33RS), and introduced to the AsPC-1 cells with a 1:1000 dilution of polybrene. The harvesting process was repeated at 6 and 24 h after the initial introduction of the 293T media to the AsPC-1 cells. At 30 h, the AsPC-1 cells were split and allowed to grow for 72 h. Live cell fluorescence imaging confirmed presence of the luciferase plasmid in the target AsPC-1 cells.

Cell sorting was performed by the Flow Cytometry Core at Memorial Sloan Kettering Cancer Center. 8×10^6 transduced AsPC-1 cells were sorted on a Becton-Dickinson Biosciences FACSymphony™ S6 via selection for red fluorescent protein (*Figure S2*). After sorting, the luciferase-expressing AsPC-1 cells (AsPC-1_{luc}) were immediately cultured and maintained in a sterile environment for downstream applications.

Generation of the PDX Models

Tumor tissue from pancreatic adenocarcinoma patients were collected under an approved IRB protocol (#14-091). Tumor tissue was immediately minced, mixed (50:50) with MatriGel (Corning; New York, NY, USA) and implanted subcutaneously in six-to-eight week old female

NSG mice (Jackson Laboratory; Bar Harbor, ME, USA) to generate patient-derived xenografts (PDX) as previously described [1].

SUPPORTING FIGURES

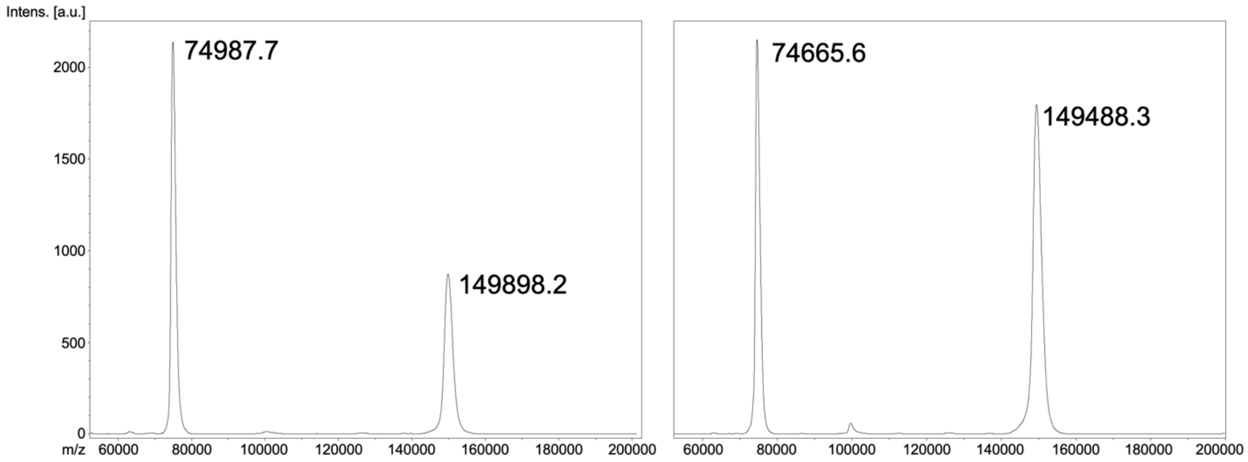

Figure S1. Representative MALDI-ToF spectra of D2101 and DFO-D2101.

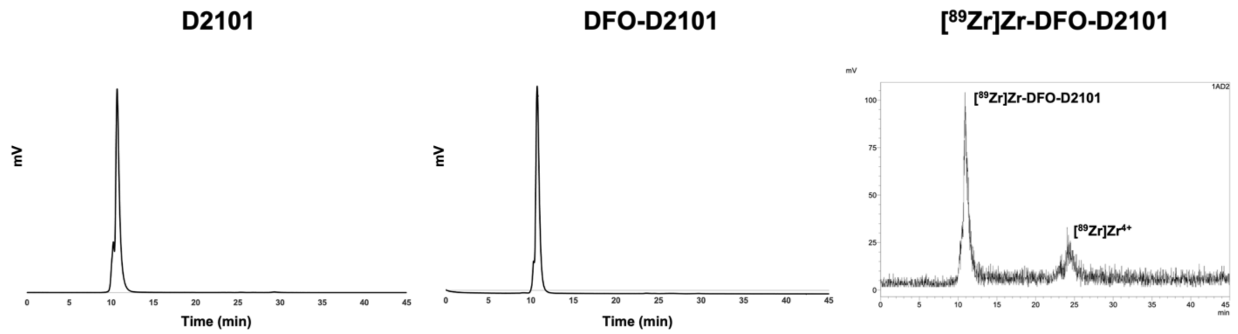

Figure S2. Size-exclusion HPLC chromatograms of D2101 (left) and DFO-D2101 (middle) as well as a radio-HPLC chromatogram of [^{89}Zr]Zr-DFO-D2101 (right).

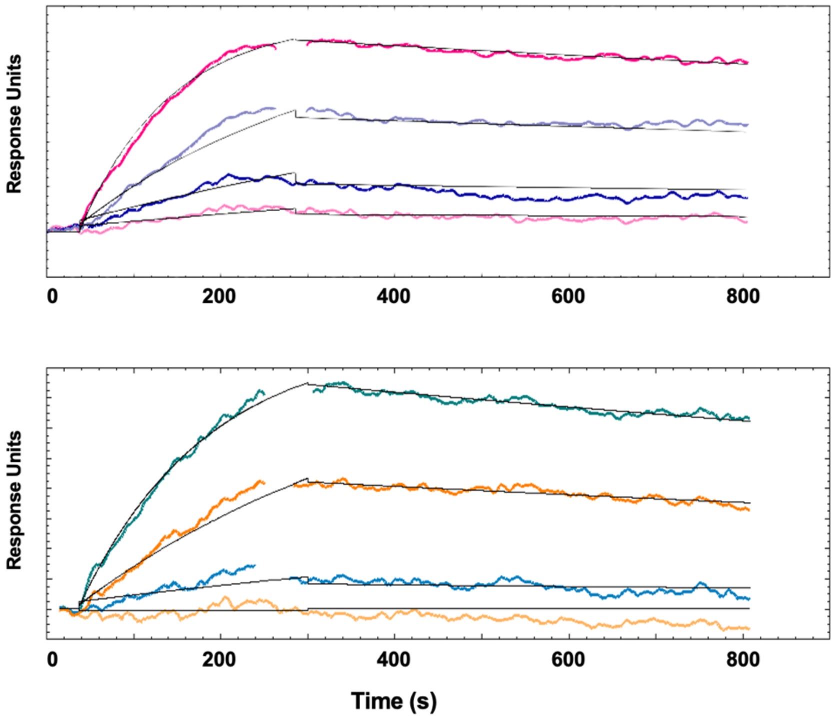

Figure S3. Surface plasmon resonance spectra of D2101 (top) and DFO-D2101 (bottom) with recombinant CDH17.

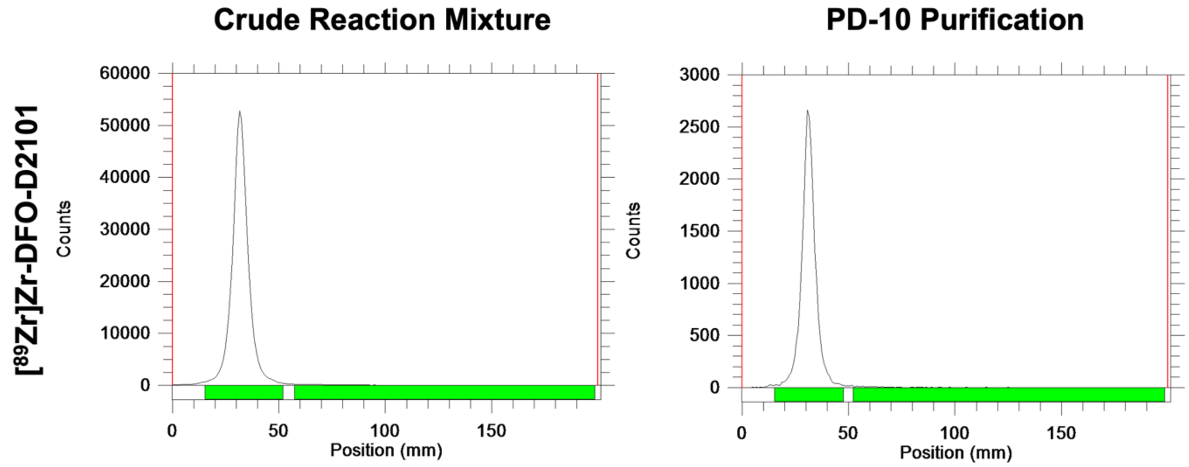

Figure S4. Radio-iTLC chromatograms of [^{89}Zr]Zr-DFO-D2101 before and after purification using a PD-10 gel filtration column.

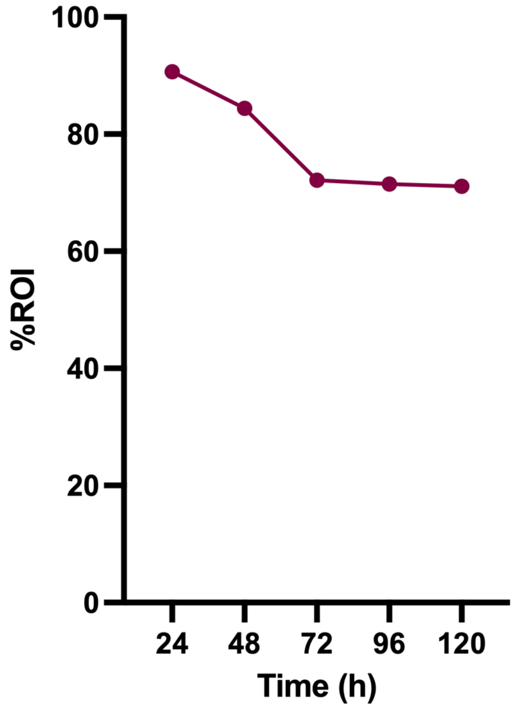

Figure S5. The stability study of [^{89}Zr]Zr-DFO-D2101 in mouse serum over the course of 5 d. Radio-iTLC measurements were taken in triplicate every 24 h.

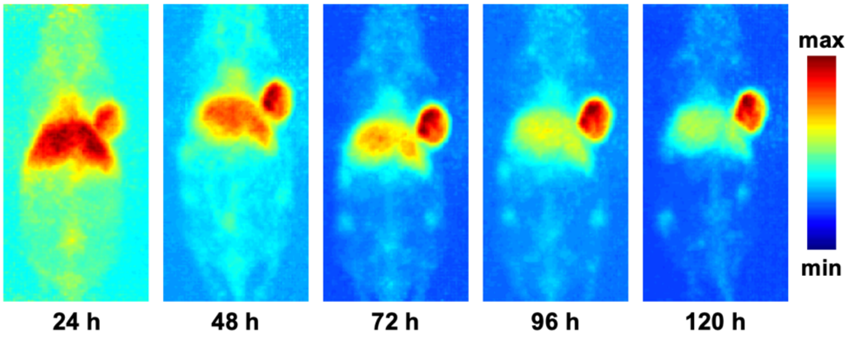

Figure S6. Maximum intensity projection PET images acquired 24, 48, 72, 96, and 120 h after the administration of [^{89}Zr]Zr-DFO-D2101 [3.7 MBq (20 μg) in 100 μL PBS] to mice bearing subcutaneous AsPC-1 xenografts.

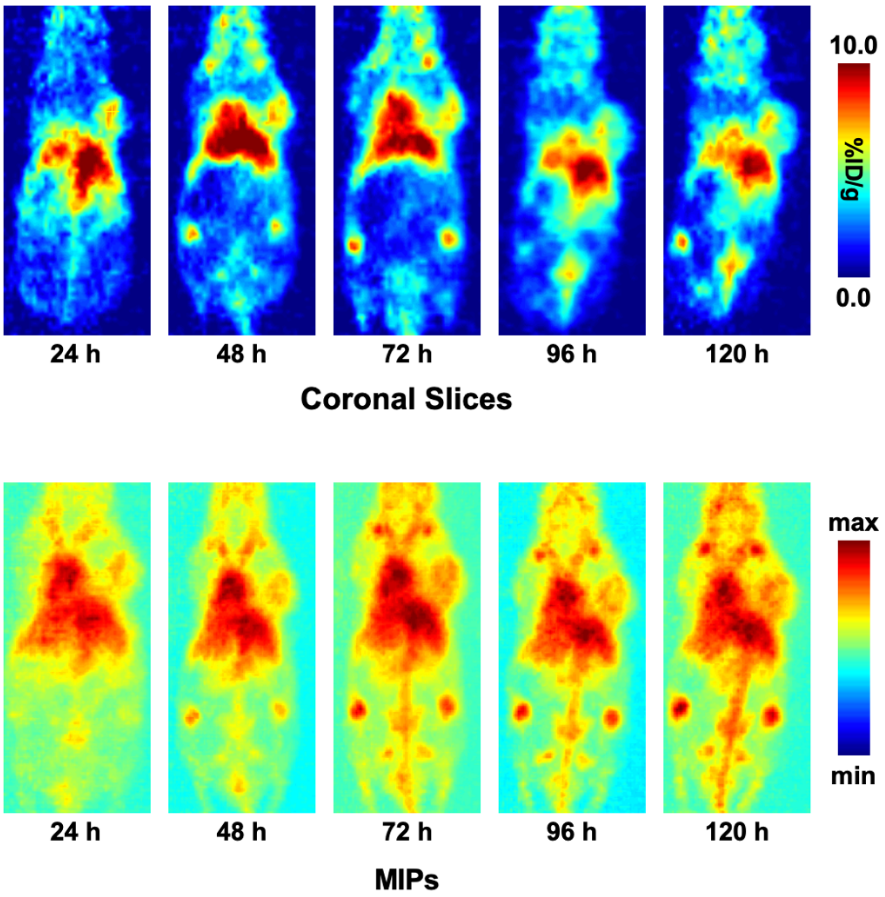

Figure S7. Coronal slices and maximum intensity projection PET images acquired 24, 48, 72, 96, and 120 h after the administration of [^{89}Zr]Zr-DFO-IgG [3.7 MBq (20 μg) in 100 μL PBS] to mice bearing subcutaneous AsPC-1 xenografts.

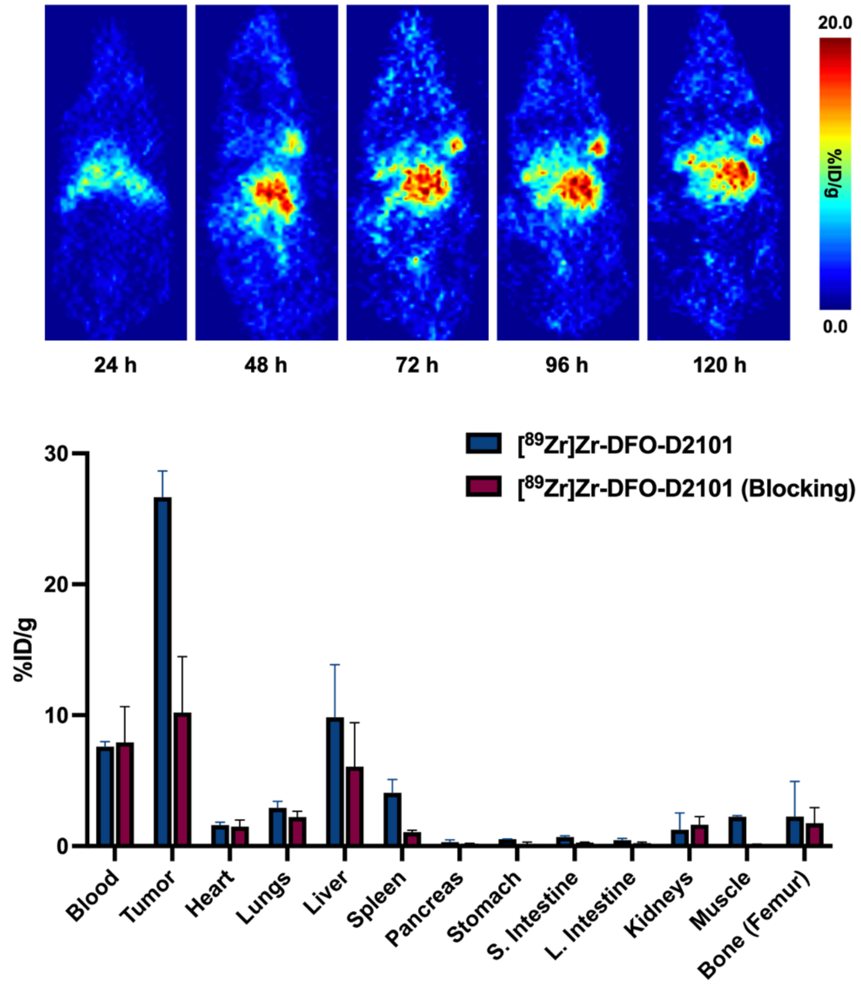

Figure S8. (Top) Coronal PET images acquired 24, 48, 72, 96, and 120 h after the administration of [^{89}Zr]Zr-DFO-D2101 [2.96 MBq (20 μg) in 100 μL PBS] with an excess of unmodified D2101 (~400 μg) to mice bearing subcutaneous AsPC-1 xenografts. (Bottom) Comparison of the biodistribution data from the blocking experiment to data obtained using [^{89}Zr]Zr-DFO-D2101 alone [2.96 MBq (20 μg) in 100 μL PBS] in mice with subcutaneous AsPC-1 xenografts.

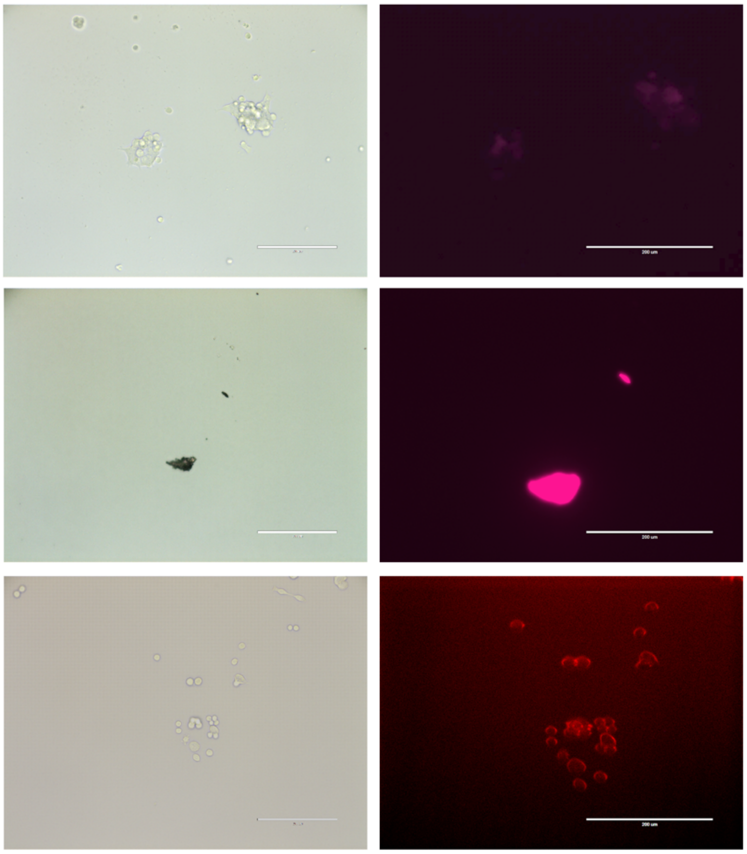

Figure S9. Brightfield (left) and fluorescent (right) images of luciferase-expressing HEK293T cells (top), luciferase-expressing AsPC-1 cells (middle), and the sorted luciferase expressing AsPC-1 cells (bottom).

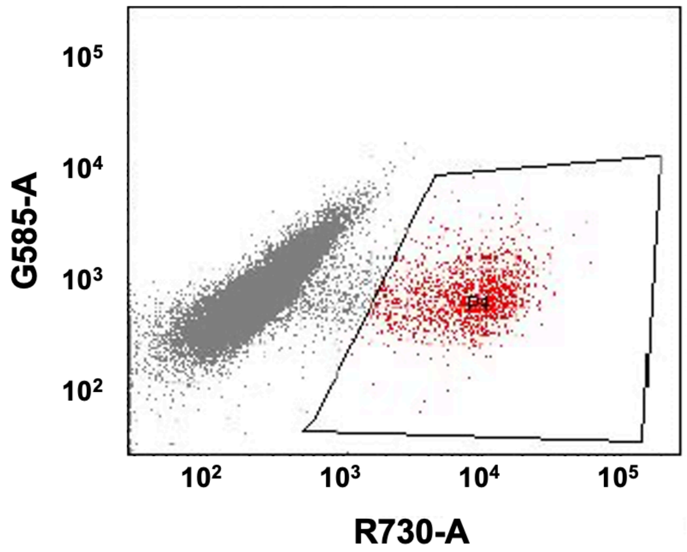

Figure S10. Fluorescence-associated cell sorting data of transduced AsPC-1 cells. Cells were selected for red fluorescent protein.

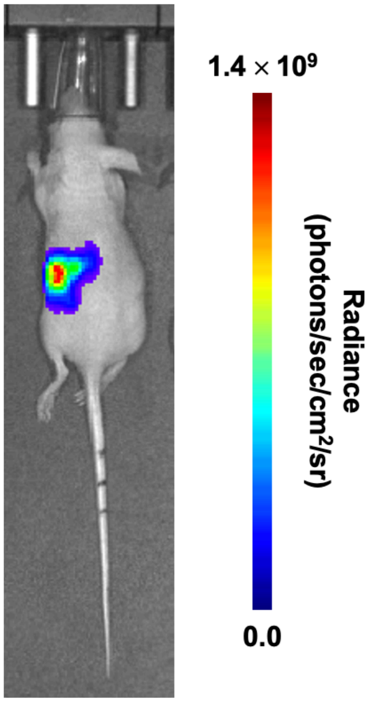

Figure S11. Representative *in vivo* bioluminescence image of an orthotopic AsPC-1_{luc} xenograft ~2 weeks after surgical implantation.

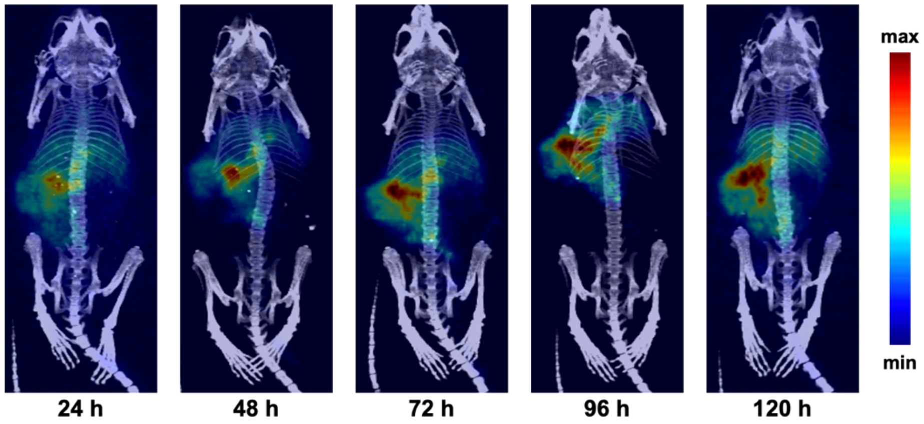

Figure S12. Maximum intensity projection PET-CT images acquired 24, 48, 72, 96, and 120 h after the administration of [^{89}Zr]Zr-DFO-D2101 [2.96 MBq (20 μg) in 100 μL PBS] to mice bearing orthotopic AsPC-1$_{luc}$ xenografts.

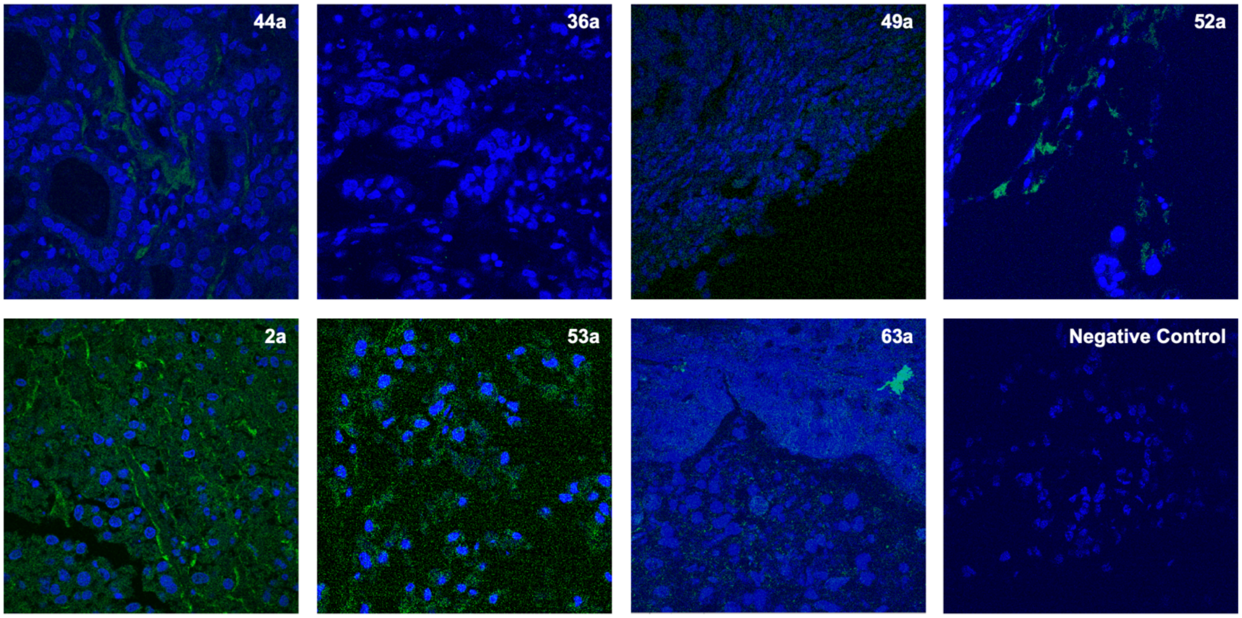

Figure S13. CDH17-targeted immunofluorescence staining of patient-derived PDAC tissues that were *not* subsequently grown in mice as PDXs. Blue: DAPI; Green: Goat anti-human IgG Alexa-488.

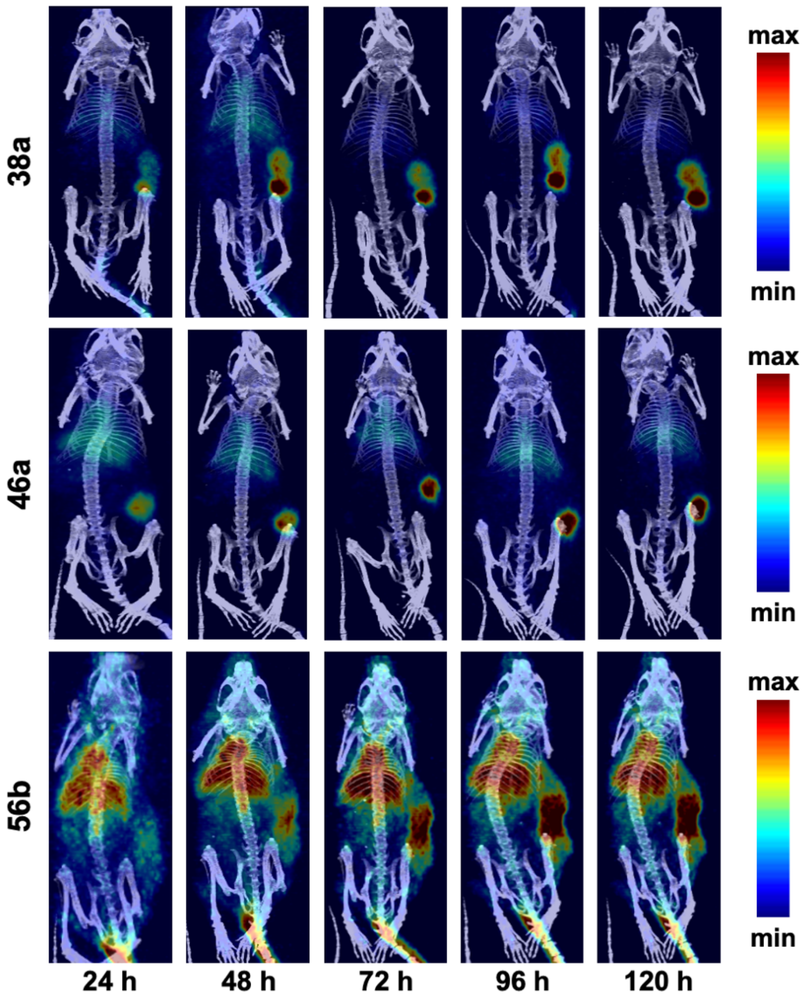

Figure S14. Maximum intensity projection PET-CT images acquired 24, 48, 72, 96, and 120 h after the administration of [^{89}Zr]Zr-DFO-D2101 [2.96 MBq (20 μg) in 100 μL PBS] to mice bearing patient-derived PDAC xenografts.

SUPPORTING TABLES

Organ	[89Zr]Zr-DFO-D2101	[89Zr]Zr-DFO-IgG	[89Zr]Zr-DFO-D2101 (Blocking)
**Blood**	7.6 ± 0.4	9.9 ± 3.3	7.9 ± 2.7
**Tumor**	26.6 ± 2.0	5.8 ± 1.3	10.2 ± 14.2
**Heart**	1.6 ± 0.2	2.7 ± 0.6	1.5 ± 0.5
**Lungs**	2.9 ± 0.5	3.0 ± 1.9	2.2 ± 0.5
**Liver**	9.8 ± 4.0	5.7 ± 2.4	6.1 ± 3.4
**Spleen**	4.1 ± 1.0	3.3 ± 1.8	1.1 ± 0.2
**Pancreas**	0.3 ± 0.2	1.2 ± 0.2	0.2 ± 0.1
**Stomach**	0.5 ± 0.0	0.7 ± 0.2	1.2 ± 0.1
**Small Intestine**	0.7 ± 0.1	1.2 ± 0.1	0.3 ± 0.1
**Large Intestine**	0.5 ± 0.1	0.8 ± 0.2	0.3 ± 0.1
**Kidneys**	1.2 ± 1.3	0.5 ± 0.1	1.7 ± 0.6
**Muscle**	2.2 ± 0.1	3.8 ± 1.3	0.9 ± 0.1
**Bone (Femur)**	2.3 ± 2.7	5.8 ± 2.9	1.8 ± 1.2
**Skin**	1.4 ± 0.2	3.1 ± 1.6	1.4 ± 1.0

Table S1. *Ex vivo* biodistribution data collected 120 h after the administration of [^{89}Zr]Zr-DFO-D2101 [3.7 MBq (20 μg) in 100 μL PBS], [^{89}Zr]Zr-DFO-IgG [3.7 MBq (20 μg) in 100 μL PBS], and [^{89}Zr]Zr-DFO-D2101 [2.96 MBq (20 μg) in 100 μL PBS with an excess of unmodified D2101 (~400 μg)] to mice bearing subcutaneous AsPC-1 PDAC xenografts.

Organ	$[^{89}Zr]$Zr-DFO-D2101
**Blood**	3.0 ± 1.0
**Tumor**	36.3 ± 20.3
**Heart**	1.0 ± 0.7
**Lungs**	1.4 ± 0.5
**Liver**	15.6 ± 5.5
**Spleen**	15.9 ± 9.5
**Pancreas**	2.1 ± 2.9
**Stomach**	7.4 ± 8.2
**Small Intestine**	0.4 ± 0.2
**Large Intestine**	0.5 ± 0.3
**Kidneys**	4.5 ± 0.8
**Muscle**	0.5 ± 0.1
**Bone (Femur)**	5.3 ± 0.9
**Skin**	1.9 ± 1.1

Table S2. *Ex vivo* biodistribution data collected 120 h after the administration of $[^{89}Zr]$Zr-DFO-D2101 [2.96 MBq (20 μg) in 100 μL PBS] to mice bearing orthotopic AsPC-1 PDAC xenografts.

Model	Passage	Stage	Treatment Status
**38a**	2	IIB; T3N1M0	Untreated
**46a**	2	IB; T2N0M0	Untreated
**56b**	2	IV; T4N0M1	Treated

Table S3. Clinical information on the three patient-derived xenograft models used in the study.

Organ	38a	46a	56b
**Blood**	11.3 ± 2.0	24.3 ± 1.8	15.8 ± 5.3
**Tumor**	69.1 ± 24.7	61.7 ± 20.0	17.2 ± 4.3
**Heart**	4.7 ± 0.1	5.4 ± 0.3	3.9 ± 1.9
**Lungs**	5.7 ± 2.6	10.1 ± 4.6	9.7 ± 2.0
**Liver**	9.8 ± 4.2	21.7 ± 11.8	17.7 ± 0.4
**Spleen**	5.9 ± 0.6	14.6 ± 0.0	9.1 ± 2.2
**Pancreas**	0.5 ± 0.2	1.2 ± 0.1	0.8 ± 0.2
**Stomach**	0.6 ± 0.2	0.8 ± 0.2	2.7 ± 1.2
**Small Intestine**	0.8 ± 0.1	1.6 ± 0.0	1.6 ± 0.4
**Large Intestine**	1.3 ± 0.4	2.7 ± 1.3	2.9 ± 1.1
**Kidneys**	2.8 ± 0.6	4.7 ± 1.4	5.3 ± 0.0
**Muscle**	0.6 ± 0.1	2.6 ± 2.9	0.7 ± 0.1
**Bone (Femur)**	3.9 ± 1.5	4.3 ± 3.5	3.0 ± 1.1
**Skin**	7.7 ± 1.0	6.6 ± 5.4	4.6 ± 6.5

Table S4. *Ex vivo* biodistribution data collected 120 h after the administration of [^{89}Zr]Zr-DFO-D2101 [2.96 MBq (20 μg) in 100 μL PBS] to mice bearing patient-derived PDAC xenografts.

Tumor-to-Organ Ratio	Subcutaneous	Orthotopic	38a PDX	46a PDX	56b PDX
**Blood**	3.5 ± 0.4	11.6 ± 2.5	6.0 ± 1.1	2.6 ± 1.0	1.1 ± 0.1
**Heart**	16.6 ± 0.9	40.0 ± 9.4	14.7 ± 5.5	11.6 ± 4.3	4.7 ± 1.2
**Lungs**	9.2 ± 0.9	30.8 ± 25.9	12.4 ± 1.4	6.4 ± 0.9	1.8 ± 0.1
**Liver**	2.9 ± 1.0	2.2 ± 0.5	8.4 ± 6.1	3.6 ± 2.9	1.0 ± 0.3
**Spleen**	6.7 ± 1.2	2.3 ± 0.1	11.9 ± 5.3	4.2 ± 1.4	2.0 ± 1.0
**Pancreas**	108.3 ± 60.1	21.8 ± 13.5	149.1 ± 3.6	50.2 ± 18.4	24.0 ± 13.1
**Stomach**	50.5 ± 2.1	8.8 ± 7.0	113.4 ± 1.6	72.3 ± 6.1	6.7 ± 1.4
**S. Intestine**	38.2 ± 1.6	92.0 ± 13.7	93.0 ± 45.7	39.6 ± 11.9	11.4 ± 5.6
**L. Intestine**	60.5 ± 13.4	74.4 ± 10.3	54.0 ± 0.5	24.3 ± 4.3	6.6 ± 4.0
**Kidneys**	46.1 ± 48.6	7.8 ± 3.1	26.0 ± 14.4	14.3 ± 8.6	3.3 ± 0.8
**Muscle**	12.0 ± 1.4	89.8 ± 69.4	113.3 ± 17.2	75.3 ± 92.1	26.4 ± 10.4
**Bone**	39.3 ± 46.0	6.7 ± 2.7	20.8 ± 14.7	24.6 ± 24.8	5.9 ± 0.7

Table S5. Tumor-to-healthy organ activity concentration ratios calculated using the biodistribution data collected 120 h after the administration of [^{89}Zr]Zr-DFO-D2101 to athymic nude mice bearing subcutaneous [3.7 MBq (20 µg) in 100 µL PBS] and orthotopic AsPC-1 xenografts as well as NSG mice bearing patient-derived xenografts [2.96 MBq (20 µg) in 100 µL PBS].

REFERENCES

1. Mattar M, McCarthy CR, Kulick AR, Qeriqi B, Guzman S, de Stanchina E. (2018) Establishing and maintaining an extensive library of patient-derived xenograft models. Front Oncol 8:19.
